# Second Branchial Cleft Cyst: A Case Report

**DOI:** 10.7759/cureus.31815

**Published:** 2022-11-23

**Authors:** Zayd Berrerhdoche, Azeddine Lachkar, Drissia Benfadil, Fahd Elayoubi

**Affiliations:** 1 Department of Otolaryngology, Head and Neck Surgery, University Hospital Center Mohammed VI, Mohammed First University, Faculty of Medicine and Pharmacy, Oujda, MAR; 2 Department of Otolaryngology, Head and Neck Surgery, University Hospital Center Mohammed VI, Mohamed First University, Faculty of Medicine and Pharmacy, Oujda, MAR

**Keywords:** amygdaloid cysts, case report, fistulas, surgery, branchial apparatus

## Abstract

Amygdaloid cysts are benign dysembryological cystic tumors that develop in the antero-lateral part of the neck; they represent 2% of laterocervical tumors of the neck; they are among the most frequent gill anomalies; they represent 6.1% to 85.2% of second cleft anomalies. They are due to the persistence of the cervical sinus during the differentiation of the branchial apparatus. They are manifested by a laterocervical swelling located at the anterior edge of the sterno-cleido-mastoid muscle. Their cystic nature is confirmed by ultrasound and CT. Treatment consists of surgical excision.

We report the case of a 33-year-old man who consulted for a huge right laterocervical swelling that had been evolving for 16 months without any other associated symptoms. An exploratory cervicotomy with an anatomo-pathological study was performed, and the histological diagnosis retained was an amygdaloid cyst without signs of malignancy.

The objective of this work is to analyze the anatomo-clinical characteristics and discuss the methods of management and the therapeutic indications of this affection.

## Introduction

Amygdaloid cysts are among the most common gill anomalies, accounting for 6.1% to 85.2% of second cleft anomalies. They are due to the persistence of the cervical sinus during the differentiation of the branchial apparatus. The usual site is the middle third of the anterior border of the sterno-cleido-mastoid muscle, but they can be located at any point from the middle constrictor muscle of the pharynx to the supraclavicular region. We report this rare case of a huge laterocervical amygdaloid cyst [1].

## Case presentation

**Figure 1:**
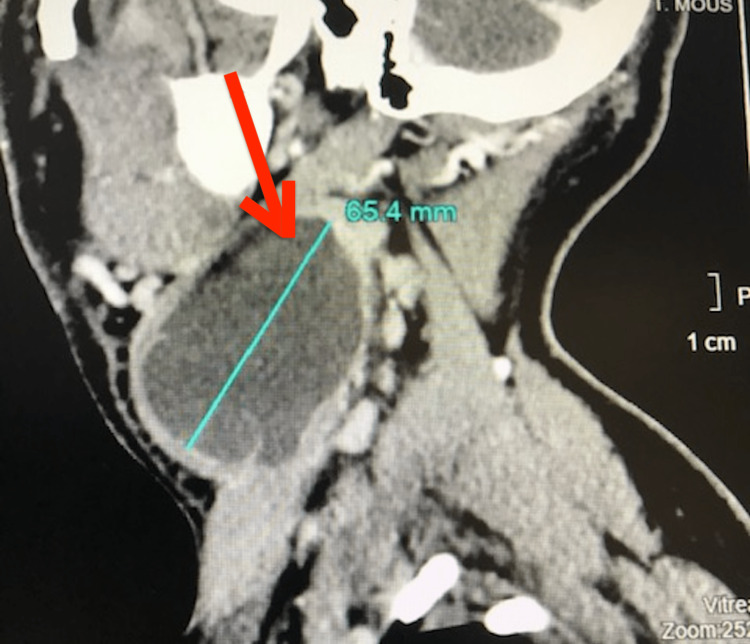
Cervico-facial CT scan injected in sagittal section showing a voluminous pseudo-cystic formation of 65/50 mm right isolated heterogeneous extending from the mandibular angle to the supraclavicular region responsible for extrinsic compression of the anterior surface of the internal jugular vein without satellite lymphadenopathy (red arrow).

A 33-year-old patient with chronic smoking and occasional alcoholism and no other notable pathological history presented for 16 months with a right laterocervical swelling that gradually increases in volume without associated otological or rhinological signs; clinical examination revealed an enormous right laterocervical tumefaction stretching from the tip of the mastoid above to the supraclavicular region below, painless, slightly mobile, non-pulsatile, measuring almost 70 mm in long axis, of renitent consistency, its full limit was impossible to specify, the adjacent skin was healthy. Examination of the oropharynx, nasopharynx, and pharyngolarynx was normal. Contrast-enhanced computed tomography objectified the presence of Figure 1.

**Figure 2:**
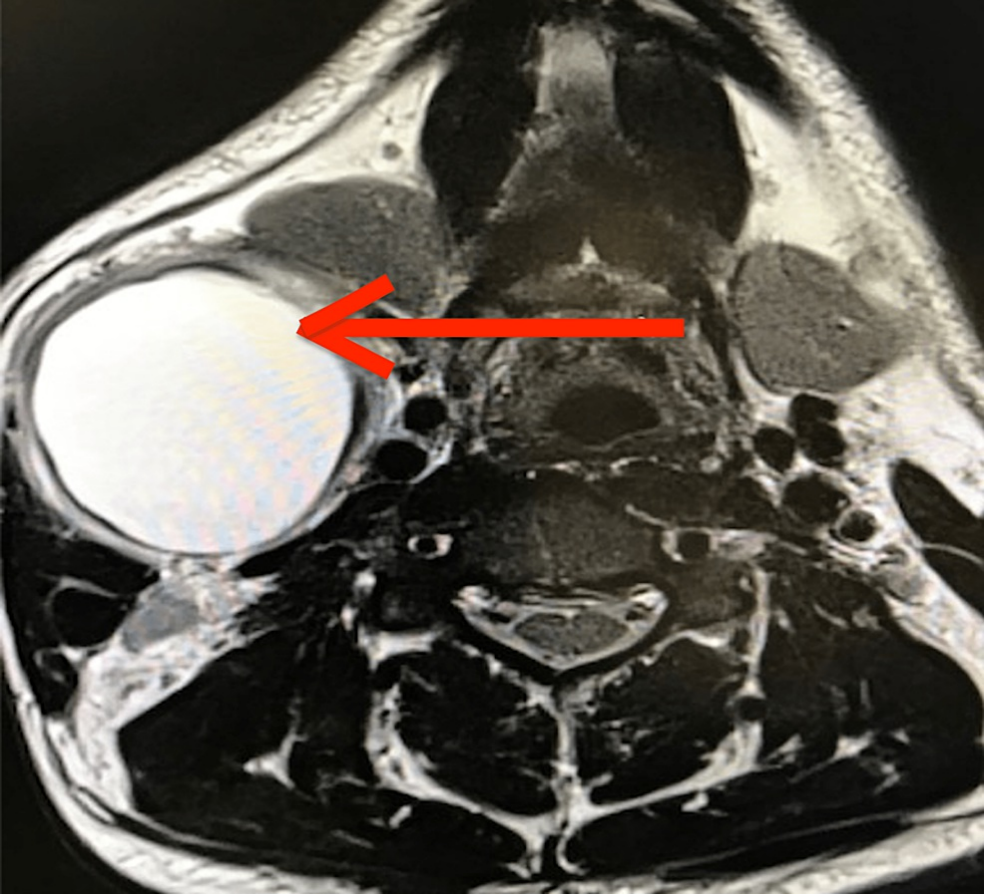
T2 cervico-facial MRI in axial section found a cystic mass under the right angulo-mandibular with a thick wall in its upper part of the same dimensions associated with gonglions in the territories of IIa and IIb suggesting a cyst of the second branchial cleft (red arrow).

The cervico-facial MRI found a cystic mass under the right angulo-mandibular wall with a thick wall in its upper part of the same dimensions associated with lymph nodes in the territories of IIa and IIb, suggesting a cyst in the second branchial cleft (Figure 2).

**Figure 3:**
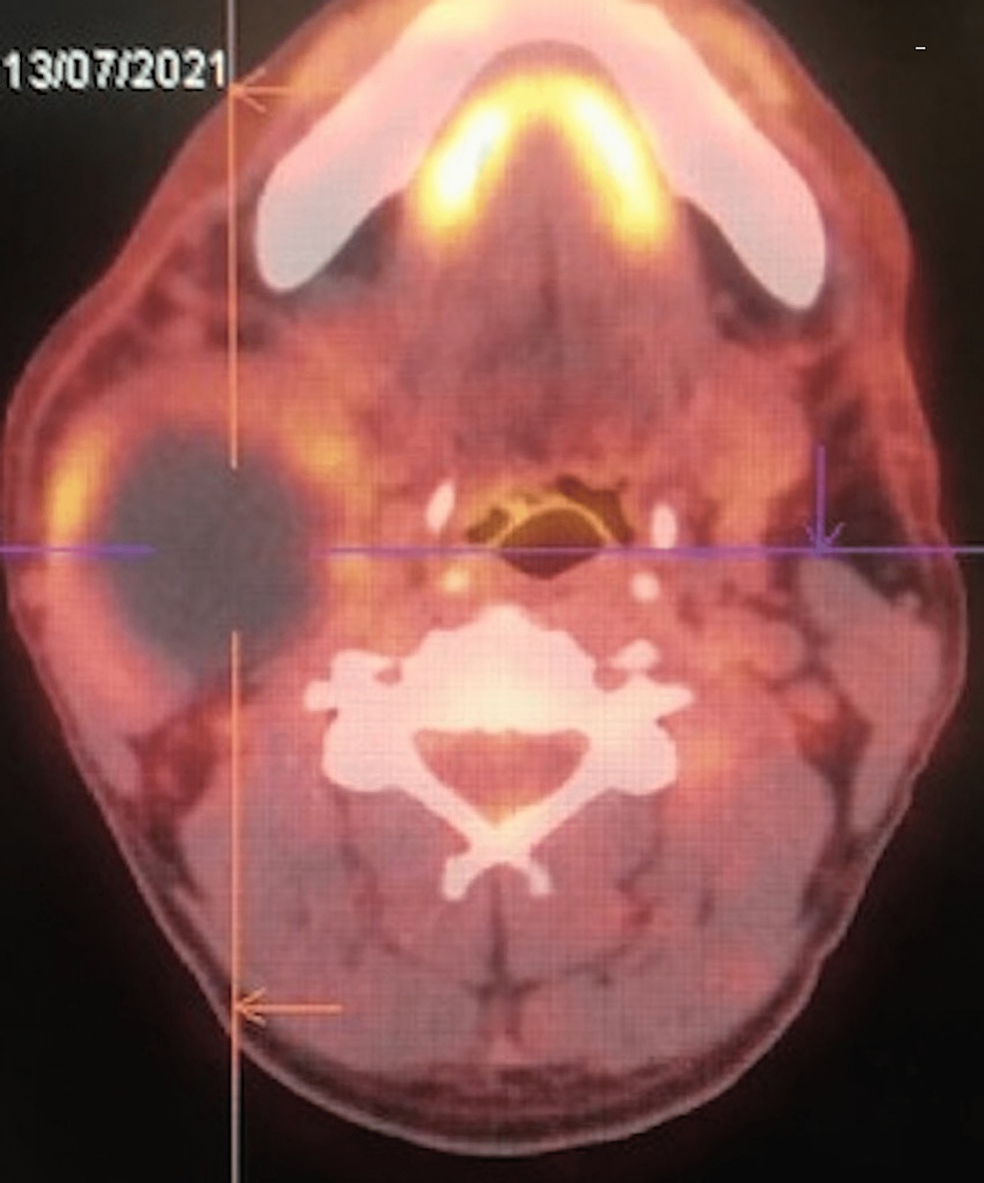
PET scanner in axial section showing a relatively intense hypermetabolic area with a necrotic center under the right angulo-mandibular measuring (49 mm × 54 mm ×69 mm).

The PET scanner (Figure 3) shows a relatively intense hypermetabolic area with a necrotic center under the right angulomandibular (49 mm × 54 mm × 69 mm) The diagnosis of a cervical cyst was retained. The patient benefited from a left cervicotomy with complete resection of the cyst; the postoperative course was simple.

**Figure 4:**
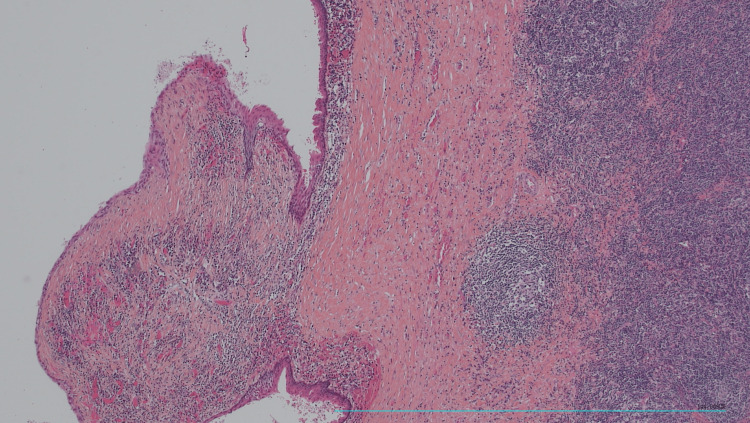
The tonsilloid cyst: lined by a squamous-type epithelium with the presence of keratin and lymphoid tissue (HE, 40×). The tonsilloid cyst: lined by a squamous-type epithelium with the presence of keratin and lymphoid tissue (HE, 40×).

Histological examination confirmed the diagnosis of an amygdaloid cyst without signs of malignancy (Figure 4).

## Discussion

Cysts and fistulas of the face and neck are uncommon and poorly understood congenital malformations. The ENT specialist must recognize these lesions early to allow for appropriate management [1,2]. Tonsillar cysts are rare benign cystic dyssembryological tumors that correspond to resorptive defects of the second branchial arch [3,4] and develop in the anterolateral part of the neck. The frequency of amygdaloid cysts in relation to second branchial arch defects varies from 6.1% to 85.2% [1,2]. The child of less than five years and between the second and third decade is the age of discovery, with two peaks of frequency. In addition, there was no gender predominance.

Clinically, a cervical sinus cyst presents as an oval kidney swelling, mobile with respect to the superficial plane, usually located near the carotid bifurcation in a subhyoid position [4,5]. The lesion is usually identified between the second and fourth decades of life, when it increases in size or becomes symptomatic [2]. The lesion may communicate externally through a narrow channel, forming a so-called external cervical fistula, the external orifice of which is located at the junction of the middle and lower thirds of the anterior border of the sternocleidomastoid muscle.

CT or MRI are particularly indicated to differentiate the lesion from other parapharyngeal tumors: hemangioma, lymphangioma, metastatic adenopathy, whose distinction with a degenerated amygdaloid cyst or an intracystic metastasis is very difficult and the confirmation remains anatomopathological after a surgical removal [1,2]. Magnetic resonance imaging (MRI) confirms the cystic nature and the proximity of the large vessels of the neck without prejudging the primary or secondary character of the malignant tonsillar cyst. The presence of a second cleft fistula should prompt a search for branchiootorenal syndrome by renal ultrasound. These cysts can be classified into four stages by Bailey [4]. We distinguish type I: superficial cyst; under the superficial cervical fascia; type II: cyst under the middle cervical fascia, in the pre-vascular region (the most common); type III: inter-vascular cyst, in the fork between the internal and external carotid arteries; and finally, type IV: intravascular cyst, between the pharyngeal wall and the carotid axis.

Histologically, the tonsillar cyst is lined by an epithelium that is most often squamous [2], but it may also be a ciliated columnar epithelium of ectodermal origin. The presence of keratin and lymphoid tissue are essential criteria for the diagnosis of a tonsillar cyst [2]. The differential diagnosis is made in children with unilocular cystic lymphangioma, lipoma, or adenopathy, especially when there is a unilateral laterocervical mass [5]. Infection is the main complication of this malformation, complicating the surgical removal of the cyst. Cervical sinus cysts can cause discomfort and bradycardia when they are located at the level of the carotid bulb, in which case cyst puncture can be proposed to improve the patient's symptoms. Malignant transformation within the branchial cyst remains exceptional [3].

## Conclusions

Amygdaloid cysts are benign dysembryologic cystic tumors developing in the anterolateral portion of the neck. They mostly result from branchial cleft abnormalities. They are due to the persistence of the cervical sinus during the differentiation of the branchial apparatus. They manifest as a laterocervical swelling in the anterior edge of the sternocleidomastoid muscle. Their diagnosis is suspected based on physical examination oriented by imaging data and confirmed by an anatomopathological examination. Therapeutic management is always surgical and should be performed as early as possible to limit the risk of inflammatory changes related to infectious episodes.
